# A Systematic Review and Meta-Analysis on the Effectiveness and Safety of Tranexamic Acid for Postpartum Haemorrhage in Patients with Haemorrhagic Disorders

**DOI:** 10.3390/diseases14010034

**Published:** 2026-01-19

**Authors:** Victor Abiola Adepoju, Abdulrakib Abdulrahim, Bukola Olanrewaju Olaniyi, Qorinah Estiningtyas Sakilah Adnani, Shankar Biswas

**Affiliations:** 1 Department of HIV and Infectious Diseases, Jhpiego Nigeria (An Affiliate of Johns Hopkins University), Abuja 900911, Nigeria; 2 Department of Medical Microbiology, Faculty of Medicine and Health Sciences, Universiti Putra Malaysia, Serdang 43400, Malaysia; abdulrakib161@gmail.com; 3 Department of Prevention, Care and Treatment, Institute of Human Virology, Lagos 101233, Nigeria; 4 Department of Public Health, Faculty of Medicine, Universitas Padjadjaran, Bandung 45363, Indonesia; qorinah.adnani@unpad.ac.id; 5 Department of Internal Medicine, Ivano-Frankivsk National Medical University, 76000 Ivano-Frankivsk, Ukraine; sb740927@gmail.com

**Keywords:** tranexamic acid, postpartum haemorrhage, bleeding disorders, von Willebrand disease, maternal health, systematic review

## Abstract

**Background:** Postpartum haemorrhage (PPH) remains the leading cause of maternal mortality globally. Women with inherited or unexplained bleeding disorders such as von Willebrand disease (VWD), factor XI deficiency (FXI), platelet function disorders, or bleeding disorder of unknown cause (BDUC) face a higher risk. While tranexamic acid (TXA) is routinely used in obstetric care, its specific efficacy and safety in these populations remain unclear. **Methods:** A systematic review and meta-analysis followed PRISMA 2020 guidelines (PROSPERO: CRD420251082349). Databases searched included PubMed, Scopus, Web of Science, and Dimensions. Studies evaluating TXA for PPH prevention or treatment in women with bleeding disorders were included. Six cohort studies (2016–2024) involving 213 deliveries met the criteria. Three contributed to a meta-analysis on primary PPH; the other three were synthesised narratively. **Results:** TXA use was associated with a 56% reduction in primary PPH risk (risk ratio 0.44; 95% CI: 0.27–0.70; *p* = 0.0007), with no observed heterogeneity (I^2^ = 0%). Because contributing cohorts were phenotypically heterogeneous (BDUC, FXI, mixed), the pooled effect reflects an average across disorders rather than disorder-specific efficacy. TXA also appeared to reduce secondary and severe PPH in some cohorts. However, bleeding occurred in 26–36% of high-risk deliveries despite prophylaxis. No maternal deaths or thromboembolic events were reported in 136 TXA-exposed cases. Attribution was complicated by concurrent use of desmopressin and platelet transfusions. Most studies had moderate to severe bias. **Conclusions:** TXA significantly lowers the risk of primary PPH in women with bleeding disorders and appears safe. Despite this, residual bleeding underscores the need for trials to optimise TXA use alongside disease-specific strategies. However, this conclusion is derived from only six observational studies with heterogeneous patient populations and co-interventions. The evidence remains preliminary and should be interpreted cautiously. TXA should be considered as part of a multimodal postpartum haemorrhage management algorithm rather than a stand-alone therapy.

## 1. Introduction

Postpartum haemorrhage (PPH) is a leading cause of maternal mortality worldwide [1,2]. It accounts for approximately 25% of maternal deaths and affects about 2–6% of all deliveries [3]. Women with pre-existing bleeding disorders (hereditary or acquired) face an even higher risk of PPH due to impaired haemostasis [2,3,4]. In conditions such as von Willebrand disease (VWD), haemophilia carriership, rare platelet function disorders (e.g., Bernard-Soulier syndrome), and fibrinolytic defects (e.g., plasminogen activator inhibitor-1 deficiency), the normal physiological adaptations of pregnancy (rise in clotting factors, increased platelet reactivity) are blunted or insufficient [5]. Consequently, these women experience both primary PPH (within 24 h of birth) and secondary PPH (24 h–6 weeks postpartum) at rates well above the general obstetric population [4]. For example, studies report primary PPH incidences of 22–44% in VWD [6,7], and 50% or more of deliveries complicated by PPH in certain platelet disorders like Bernard-Soulier syndrome [8].

Tranexamic acid (TXA), an antifibrinolytic agent, has emerged as a key intervention for PPH in the general population. TXA competitively inhibits plasminogen activation and fibrin clot breakdown, promoting haemostasis [9]. Large randomised trials in unselected obstetric patients (e.g., the WOMAN trial) demonstrated that early TXA (within 3 h of birth) significantly reduces death due to bleeding by about 30% without increasing thromboembolic events [9,10]. Prophylactic TXA administered immediately after childbirth has also been shown to decrease the incidence of PPH > 1000 mL in both vaginal and caesarean births [9]. These findings led the World Health Organization to recommend TXA for PPH treatment as part of the first-line response, given its strong safety profile and cost-effectiveness [10]. However, women with bleeding disorders were generally not separately studied in those large trials, and it remains uncertain how effective TXA is in the context of pre-existing coagulopathies or whether any unique safety considerations arise in this population.

Current management of pregnant women with bleeding disorders relies on raising deficient clotting factor levels (e.g., VWF/FVIII concentrates in VWD, platelet transfusions in platelet disorders, etc.) and close monitoring by a multidisciplinary team [5]. Despite such specialised care, studies have found that PPH still occurs in a substantial proportion of these women [5,11]. This has prompted interest in adjunct therapies like TXA to reduce bleeding further. Antifibrinolytic therapy is routinely used for heavy menstrual bleeding in bleeding disorders and is biologically plausible in the postpartum setting, as the fibrinolytic system is highly activated at placental separation [7]. Some guidelines and experts already advocate TXA prophylaxis for 7–14 days postpartum in VWD and other bleeding diatheses, aiming to prevent late PPH when factor levels fall back to baseline [5,7]. On the other hand, questions remain about the optimal timing, dosage, and duration of TXA in this context, and the evidence base has until recently been limited to small series, extrapolation from general PPH trials, and expert opinion [5,12].

In this systematic review and meta-analysis, we critically assess the effectiveness and safety of TXA in preventing and managing postpartum haemorrhage among women with inherited or unexplained bleeding disorders. Unlike general obstetric populations widely studied in large, randomised trials, these women face distinct haemostatic vulnerabilities yet remain understudied in clinical research. By focusing on conditions such as von Willebrand disease, platelet function disorders, rare coagulation factor deficiencies, fibrinolytic abnormalities, and bleeding of unknown cause (BDUC), we synthesise data from observational cohorts and real-world clinical reports to quantify the potential role of TXA in reducing PPH incidence, minimising transfusion needs, and averting adverse maternal outcomes. This review evaluates the therapeutic and prophylactic utility of TXA in this underrepresented group and also highlights critical evidence gaps related to timing, dosage, and disease-specific responses. We aim to inform clinical decision-making and guide future research and ultimately advance equity in maternal health for women with bleeding disorders who are often excluded from mainstream obstetric guidelines and interventions.

## 2. Methodology

### 2.1. Protocol Registration and Reporting Standards

We developed the review protocol in advance and registered it on 27 June 2025 with the International Prospective Register of Systematic Reviews (PROSPERO) under the registration number CRD420251082349. The methodology was designed and reported following the PRISMA 2020 checklist [13].

### 2.2. Eligibility Criteria

We applied a structured PECOS framework to guide study selection. To be eligible, studies must assess the impact of TXA for either prophylaxis or treatment of PPH, specifically in women with inherited or acquired bleeding disorders (Table 1). No language or publication date restrictions were applied.

### 2.3. Information Sources

We systematically searched four databases: PubMed, Scopus, Web of Science, and Dimensions.ai. The search covered all available years up to May 2025. Grey literature was accessed via conference proceedings, and references were traced using Google Scholar.

### 2.4. Search Strategy

Search terms combined synonyms and controlled vocabulary for the concepts of: (1) TXA, (2) postpartum haemorrhage, and (3) bleeding disorders. Boolean operators were used to achieve optimal sensitivity. The detailed database-specific queries are provided in Supplementary Table S1.

### 2.5. Selection Process

**Figure 1:**
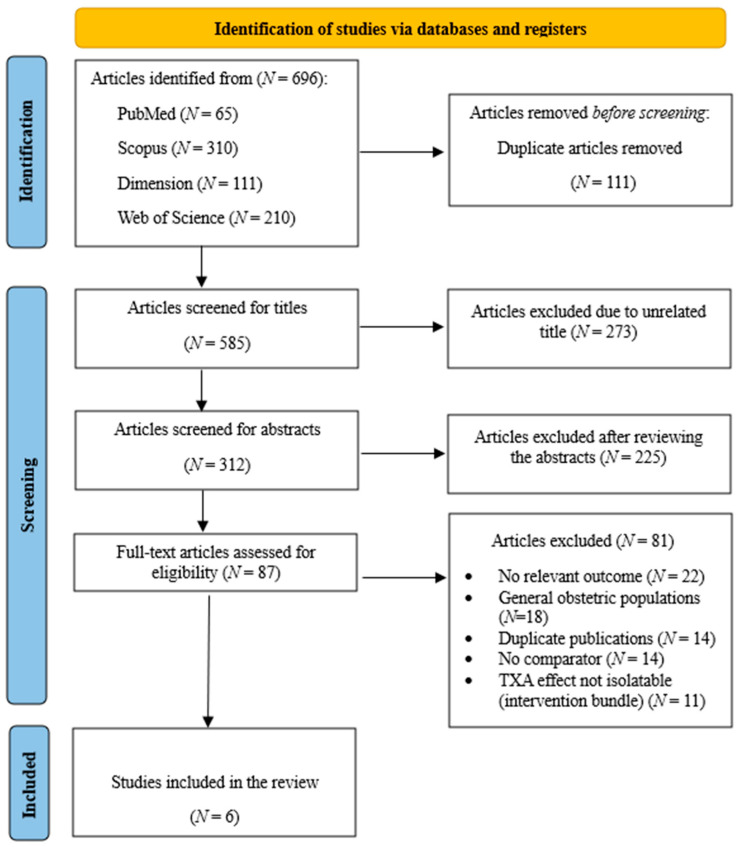
PRISMA 2020 flow diagram.

We imported all 696 records into EndNote 21 and removed 111 duplicates using exact match criteria (title + author) and fuzzy logic. After deduplication, 585 unique records remained. Two reviewers (VAA and SB) independently screened the titles and abstracts. Of these, 273 articles were excluded due to titles unrelated to PPH, TXA, or bleeding disorders, while 312 articles were identified for abstract review. Abstract screening resulted in the removal of 225 articles, including narrative reviews, studies with insufficient data, and non-human studies. The remaining 87 articles were assessed in full by two reviewers (BOO and QESA). Disagreements were resolved through discussion or, if necessary, by a third-party adjudicator (SB). After applying the eligibility criteria, six studies were included in the final synthesis. The 81 excluded full-text articles had reasons such as: no relevant outcomes, general obstetric populations, duplicate publications, absence of comparators, and inability to isolate the effect of TXA within intervention bundles. The PRISMA 2020 flowchart (Figure 1) presents the stepwise selection process and reasons for exclusion at the full-text stage. Detailed reasons for exclusion of some of the studies are provided in Supplementary Table S2 [4,6,7,8,14,15,16,17,18].

### 2.6. Data Collection Process

We designed and piloted a structured data extraction form in Microsoft Excel. Two reviewers (QESA and SB) independently extracted all critical variables and reconciled discrepancies by consensus. The following data were captured: Author name and year of publication, study design, country, bleeding disorder type and diagnostic criteria, how TXA was used (prophylaxis vs. treatment) and dose/route, comparative characteristics, outcome definition, whether the study meets sample size and reason for eligibility (Table 2).

### 2.7. Risk of Bias Assessment

We evaluated all studies using the ROBINS-I tool for observational designs [19]. Two reviewers (VAA and SB) independently applied domain-level assessments: confounding, participant selection, intervention classification, deviations from intended interventions, missing data, outcome measurement, and selective reporting. Ratings were classified as low, moderate, severe, or critical risk.

### 2.8. Data Items and Outcome Measures

For all included studies, we extracted event counts and total number of women for the following dichotomous outcomes: (1) Primary PPH; (2) Severe PPH (≥1000 mL blood loss); (3) Secondary PPH (24 h to 6 weeks postpartum); (4) Blood transfusion; (5) Invasive haemostatic procedures; (6) Venous thromboembolism (VTE) and (7) Maternal death. The investigators used the risk ratio (RR) to estimate the effect at 95% confidence intervals.

### 2.9. Data Analysis

Effect estimates from included studies were synthesised narratively due to clinical and methodological heterogeneity. Where comparable data were available, we calculated pooled odds ratios and 95% confidence intervals using the Online Meta-Analysis Calculator (https://metaanalysisonline.com/). We used a random-effects model to account for between-study variability. We anticipated clinical heterogeneity because included cohorts encompassed von Willebrand disease, factor XI deficiency, platelet function disorders, and bleeding disorder of unknown cause. We therefore pre-specified subgroup analyses by disorder class (fibrinolytic/coagulation/platelet) and by TXA timing (prophylactic vs. therapeutic). However, insufficient, non-uniform reporting precluded these analyses. Consequently, pooled estimates should be interpreted as an average adjunctive effect across mixed phenotypes. Heterogeneity was evaluated visually (overlap of confidence intervals) given the small number of studies. No funnel plot or Egger’s test was performed due to the limited number of studies (<10).

**Table 2:** Summary of included studies.

S/N	Author and Year of Publication	Study Design	Country	Bleeding-Disorder Cohort (Women/Pregnancies)	How TXA Was Used †	Comparator/Reference Arm	Key Maternal Bleeding Outcomes Reported	Meets Size Criterion	Primary Reason Eligible
1	Naveed K. (2020) [20]	Retrospective cohort	Canada (multi-centre haemophilia clinics)	VWD, FXI-def., BSS, Glanzmann, BDUC (22/29)	1 g IV q8 h × 24 h → 1 g PO TID 3–5 d (prophylaxis)	Historical and contemporaneous deliveries managed without TXA	PPH ≥ 1000 mL; transfusion; surgical/uterine interventions	≥5	Direct TXA-vs-no-TXA analysis in inherited-bleeding-disorder cohort (researchgate.net)
2	Gerber et al. (2019) [21]	Prospective case-series	US tertiary centre	Moderate/severe FXI deficiency (28/32)	1 g IV at cord-clamp ± 1 g PO TID × 3 d (prophylaxis and rescue)	Same cohort deliveries with neuraxial anaesthesia without TXA	Primary and secondary PPH; TXA-related adverse events	≥5	TXA was explicitly documented; bleeding outcomes stratified by TXA use (researchgate.net)
3	Verghese et al. (2017) [22]	10-year case series,	UK obstetric-haematology clinic	FXI deficiency (25/67)	1 g IV intra-op then 1 g PO TID × 3 d (routine prophylaxis)	Same cohort pre-2007 protocol that did not include TXA	PPH > 1000 mL; neuraxial feasibility; transfusion	≥5	Largest FXI pregnancy series; TXA regimen and outcomes described (clinmedjournals.org)
4	Castle et al. (2022) [23]	Multi-centre retrospective cohort	United Kingdom	Bleeding-disorder-of-unknown-cause (BDUC) (36/54)	1 g IV at delivery ± oral course (prophylaxis) OR 1–2 g IV for therapeutic PPH	Pregnancies with no haemostatic prophylaxis	Primary and secondary PPH; readmission; TXA AEs	≥5	Separate outcome data for TXA vs. no-TXA in BDUC cohort (ouci.dntb.gov.ua)
5	Berkowitz C. (2024) [24]	Prospective cohort	USA (periprocedural study—maternity subset)	BDUC (19 childbirths)	1 g IV at cord-clamp ± 1 g PO TID × 5 d (prophylaxis)	Procedures/deliveries where only DDAVP/other agents were used	PPH ≥ 1000 mL; transfusion; thrombo-embolism	≥5	Delivery subset meets population and exposure criteria; TXA effect separable (thieme-connect.com)
6	Hawke A. (2016) [11]	Retrospective cohort (single centre)	Canada	Mixed inherited disorders—VWD, LVWF, platelet-function defects, haemophilia carriers, FXI-def. (33/62)	Post-partum TXA (oral/IV 1 g TID for ≈ 7 d) in 34% of pregnancies (prophylaxis for secondary PPH)	Identical cohort pregnancies without TXA	Secondary PPH: 0% with TXA vs. 15% without; prolonged PP bleeding (6 wk): 10% vs. 30% (*p* = 0.049); severe PPH needing intervention: 5% vs. 20%; no thrombosis	≥5	Direct comparison of postpartum TXA vs. no-TXA within identical disorder spectrum (researchgate.net)

† Typical obstetric dose unless otherwise stated; “prophylaxis” = given routinely to avert PPH, “therapeutic” = given in response to an active bleed. TID, three times daily; IV, intravenous; LVWF, low von Willebrand factor; BDUC, bleeding disorder of unknown cause; USA, United States of America; UK, United Kingdom; PO, per os (oral administration); DDAVP: 1-desamino-8-D-arginine vasopressin.

### 2.10. Assessment of Reporting Bias

Due to the few included studies, we did not conduct Egger’s test or Harbord’s regression. Instead, we visually inspected a funnel plot for asymmetry based on the primary outcome.

## 3. Results

### 3.1. Study Selection and Characteristics

The study included six observational cohort studies that met the inclusion criteria. Three studies [20,23,24], reported arm-level comparative data on primary postpartum haemorrhage (PPH) within 24 h and were eligible for meta-analysis. The remaining three studies, [11,21,22], were retained for qualitative synthesis due to the absence of extractable or comparable denominators.

The six included studies were conducted in high-income settings (Canada, UK, USA), published between 2016 and 2024, and involved women with inherited or unexplained bleeding disorders. These disorders included VWD, platelet function disorders (PFD), FXI, and bleeding disorders of unknown cause (BDUC). Study designs included retrospective cohort studies (four studies), prospective observational studies (one study), and one case series. TXA was used either prophylactically (oral or intravenous) during or after delivery or therapeutically in response to active bleeding. Comparator arms included studies reporting pregnancies without TXA exposure or managed expectantly. Outcomes varied but generally included primary PPH (within 24 h), secondary PPH (24 h–6 weeks), severe PPH (≥1000 mL), and safety indicators such as maternal mortality or thromboembolism (Table 1). In the three studies contributing to the meta-analysis of primary PPH, BDUC and FXI deficiency comprised a substantial share of TXA-exposed deliveries [22,23], whereas Naveed 2020 [20] contributed a smaller, mixed-disorder cohort. This distribution indicates that the pooled estimate likely reflects phenotypes with prominent fibrinolysis/unknown cause more than isolated platelet or severe factor-deficiency states.

Across studies, TXA was often administered as part of a broader prophylactic strategy, in Berkowitz et al. [24], perioperative platelet transfusion was used in 26% of women with BDUC undergoing major procedures, while desmopressin and platelet infusions were also noted in Castle et al. [23]. These co-interventions highlight real-world complexity in attributing outcomes to TXA monotherapy. Across the included cohorts, the haemostatic context varied widely. Gerber et al. [21] and Naveed et al. [20] described postpartum TXA given as the sole antifibrinolytic therapy without concurrent factor replacement or DDAVP, whereas Hawke et al. [11] and Castle et al. [23], reported that TXA was routinely combined with desmopressin, VWF/FVIII concentrates, platelet transfusions or fresh-frozen plasma as part of multimodal prophylaxis. Berkowitz et al. [24], noted that approximately one-quarter of BDUC patients received perioperative platelet transfusion alongside TXA, and Setty et al. [25], highlighted cases of FXI deficiency managed successfully with TXA alone. Only a minority of studies therefore examined TXA monotherapy and most of these administered TXA within broader haemostatic packages, precluding formal subgroup analyses and underscoring the need for systematic reporting of concomitant treatments in future research.

Furthermore, the severity of bleeding disorders varied but was seldom documented. Baseline VWF or FXI levels, VWD subtype, or platelet aggregation results were generally absent from reports, making it impossible to distinguish mild from severe phenotypes. Without consistent severity data, interpretations of TXA efficacy across the spectrum, from mild to severe coagulopathies, remain speculative and limit generalizability.

In addition, delivery mode appeared to influence bleeding risk; Hawke et al. [11] found that vaginal births were significantly more likely to result in immediate PPH compared to caesarean births (*p* < 0.042), suggesting mode of childbirth may act as an effect modifier.

### 3.2. Quantitative Synthesis: Primary Postpartum Haemorrhage (≤24 h)

#### 3.2.1. Effectiveness of TXA in Preventing Primary PPH

**Figure 2:**
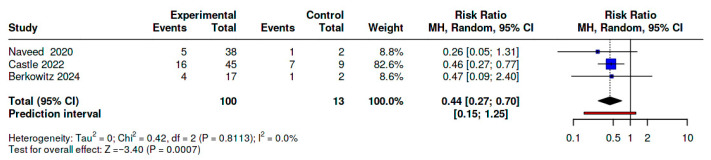
Forest Plot of TXA vs. No TXA for Primary PPH (3-Study Analysis [20,23,24]). Blue boxes indicate studies or data sources that met the eligibility criteria and were included in the quantitative synthesis, while red boxes represent records that were excluded after full-text review. Solid lines depict the primary analytical pathway used in the meta-analysis, whereas dashed lines indicate secondary or sensitivity analyses. Rectangular nodes represent aggregated analytical steps, diamonds for comparative analyses .

Three studies provided arm-level data for meta-analysis of primary PPH [20,23,24]. These studies included 100 deliveries exposed to TXA and 13 comparator deliveries. A random-effects Mantel–Haenszel model yielded a pooled risk ratio (RR) of 0.44 (95% CI: 0.27–0.70; *p* = 0.0007), indicating a 56% reduction in the relative risk of primary PPH with TXA administration. Statistical heterogeneity was negligible (I^2^ = 0%; τ^2^ = 0), indicating the consistency of the findings. A fixed-effect model produced an identical RR (0.44), reinforcing the stability of the result (Figure 2).

#### 3.2.2. Sensitivity Analyses

**Figure 3:**
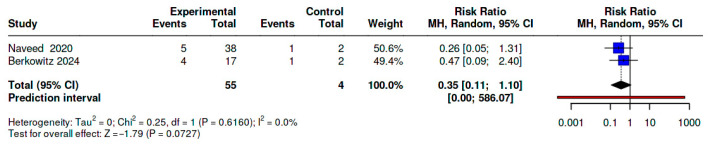
Forest Plot of TXA vs. No TXA for Primary PPH (2-Study Analysis [20,24], excluding Castle et al. [23]). Different shapes represent distinct study designs included in the analysis (e.g., diamonds for comparative analyses). Blue markers denote effect estimates included in the primary analysis, while red markers indicate estimates excluded from pooling or used only in sensitivity analyses. Dotted or dashed lines illustrate comparative or stratified analytical pathways across subgroups.

In a leave-one-out analysis excluding Castle 2022 (BDUC-dominant) [23], the pooled RR remained <1 with consistent direction of benefit but became imprecise (RR 0.35; 95% CI 0.11–1.10; *p* = 0.073), reflecting reduced information rather than reversal of effect. Excluding either Naveed et al. [20] or Berkowitz et al. [24] yielded near-identical RRs (≈0.46), suggesting no single study drove the observed association (Figure 3).

**Figure 4:**
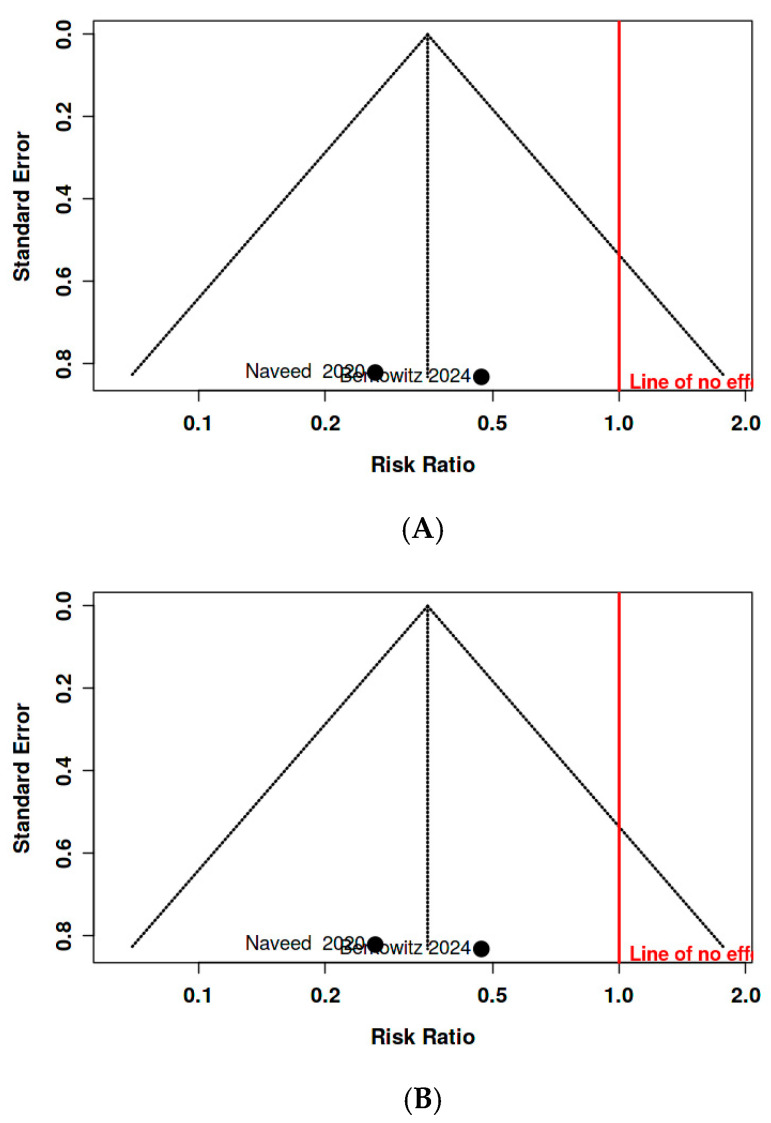
(**A**) Funnel Plot of Publication Bias for TXA vs. No TXA in. Preventing Primary PPH (All 3 Studies Included). (**B**) Funnel Plot of Publication Bias for TXA vs. No TXA in Preventing Primary PPH (Naveed et al. [20] and Berkowitz et al. [24], excluding Castle et al. [23]).

Publication Bias: Funnel plot inspection (Figure 4A) showed no apparent asymmetry, but the plot was underpowered due to the limited number of studies (*n* = 3). Egger’s regression was not performed. After excluding Castle et al. [23], a two-point funnel plot (Figure 4B) was uninterpretable.

### 3.3. Secondary Outcomes (Narrative Synthesis)

#### Secondary PPH (>24 h to 6 Weeks) and Severe PPH (≥1000 mL)

Only Hawke et al. [11], provided stratified data on secondary PPH. The use of TXA postpartum was associated with fewer secondary bleeding episodes than no TXA (7/36 vs. 11/26), yielding a risk ratio of approximately 0.46 (95% CI: 0.21–1.01). However, pooling was not feasible due to a lack of replication in other studies. Castle et al. [23] reported a RR of 0.70 (95% CI: 0.18–2.66) for severe PPH among women receiving TXA, whereas Berkowitz et al. [24], documented one event in 17 deliveries managed using TXA versus one in two comparator deliveries. Both studies reported persistent PPH in 26–36% of women despite prophylactic use, underscoring the need for adjunctive interventions or refined targeting strategies.

### 3.4. Safety Outcomes

Across the four studies reporting safety outcomes [11,20,23,24], no maternal deaths or thromboembolic events were reported among 136 TXA-exposed and 39 comparator childbirths. In Naveed et al. [20]. Despite universal TXA use during labour or postpartum, no VTE events were observed in 38 treated patients, reinforcing the favourable safety profile of TXA in this population.

### 3.5. Result of Risk of Bias Assessment

Using the ROBINS-I tool, all four comparative studies were rated at either “moderate” or “serious” risk of bias due to limitations such as confounding, selection bias, and outcome measurement. Naveed et al. [20] and Berkowitz et al. [24] were downgraded for having extremely small or unbalanced control arms. Hawke et al. [11] further rated “serious” due to subjective outcome definitions and lack of blinding (Table 3). Verghese et al. [22] and Gerber et al. [21] were not assessed due to insufficient comparator data.

### 3.6. Subgroup and Sensitivity Analyses

The planned subgroup analyses could not be executed. None of the studies stratified outcomes by timing of TXA administration (prophylactic vs. therapeutic) nor provided disaggregated data for TXA monotherapy versus combination regimens with desmopressin or platelet transfusion. In addition, disease-specific stratification (e.g., by VWD subtype or FXI level) was not possible due to small sample sizes and mixed cohorts. Nonetheless, individual studies reported real-world adjustments such as the use of fresh frozen plasma in FXI deficiency or platelet transfusion in BDUC, which varied across patients and childbirth modes [21,24].

## 4. Discussion

This systematic review offers compelling early evidence that TXA significantly reduces the risk of primary PPH among women with inherited or unexplained bleeding disorders. The pooled analysis from three eligible observational studies yielded a relative risk of 0.44 (95% CI: 0.27–0.70), suggesting a 56% reduction in PPH incidence when TXA was administered. This effect remained consistent across both fixed and random effects models and was unaccompanied by statistical heterogeneity, reinforcing the stability of the observed association. This effect was demonstrated despite the presence of adjunctive interventions such as platelet transfusions or desmopressin in some cohorts, and across varying childbirth modes while vaginal birth was identified as an independent PPH risk factor [11]. The consistency of benefit, coupled with a reassuring absence of thromboembolic events even among high-TXA exposure cohorts, lends clinical relevance to these findings and warrants integration into peripartum planning for this high-risk population. Our analytic set was a composite of bleeding-disorder phenotypes with distinct pathophysiology. Because TXA principally attenuates fibrinolysis, larger effects would be expected in BDUC/hyperfibrinolytic states or in FXI deficiency when fibrinolysis is unopposed, with potentially smaller effects where primary haemostasis (platelet function disorders) or severe factor deficiency predominates. The numerical contribution of BDUC/FXI cohorts to our meta-analysis therefore may inflate the average benefit relative to platelet or severe factor-deficiency disorders. Conversely, heterogeneity may also dilute benefits if TXA-responsive and TXA-non-responsive phenotypes are pooled. Given the foregoing, our pooled RR should be viewed as an average adjunctive effect, not a disorder-specific estimate of efficacy.

The observed benefit aligns with the broader body of literature supporting the antifibrinolytic effects of TXA in obstetrics. The ASH/ISTH/NHF/WFH 2021 clinical practice guidelines on von Willebrand disease provide formal support for this approach. The guidelines recommend the use of postpartum tranexamic acid (25 mg/kg TID for 10–14 days) in women with type 1 von Willebrand disease or low von Willebrand factor levels, including extended prophylaxis to reduce the risk of secondary postpartum haemorrhage, and affirming its safety during breastfeeding [26]. Although large-scale trials such as the WHO WOMAN trial have established the role of TXA in treating established PPH [25]. This review addresses a distinct knowledge gap: the prophylactic use of TXA in women who are genetically or clinically predisposed to bleeding. This population, often overlooked in conventional PPH prevention protocols, may derive disproportionate benefit from TXA, given their impaired haemostatic reserve.

Several clinical observations lend real-world credence to the review findings. For instance, the perioperative case series by Setty and colleagues illustrates how TXA successfully prevented excessive blood loss in three women with factor XI (FXI) deficiency undergoing high-risk surgeries, including caesarean delivery [25]. The study reported that none of these patients required factor replacement or transfusion. Their experiences reflect the potential of TXA to serve not only as an adjunct but, in select cases, as a standalone prophylactic measure in individuals with moderate bleeding phenotypes.

Further corroborating the role of TXA, the Vienna Bleeding Biobank (VIBB) has illuminated the clinical burden of bleeding disorder of unknown cause (BDUC), a diagnosis affecting over 60% of patients referred for abnormal bleeding, yet lacking a molecular signature [27]. Despite their diagnostic ambiguity, BDUC patients present with bleeding risks similar in severity to those with VWD or platelet function disorders (PFDs). For these individuals who are often managed empirically, TXA is as a crucial option and provides a safe and pragmatic approach to reducing the risk of childbirth-associated haemorrhage when other targeted therapies are unavailable or inappropriate.

One of the principal strengths of this review lies in its focus on a narrowly defined, high-risk population. Prior meta-analyses have often diluted treatment effects by pooling heterogeneous obstetric populations. In contrast, this synthesis isolates a group of women for whom bleeding risk is not only elevated but frequently underestimated. The review examines the effect of TXA in this subset and provides focused insights that may be masked in more generalised analyses. From a clinical perspective, the findings underscore the value of early TXA administration in women with diagnosed bleeding disorders, particularly those with VWD and FXI deficiency, where factor levels may either fail to rise during pregnancy or decline precipitously after childbirth. In the absence of other reliable predictors of haemorrhage in this population, prophylactic TXA may serve as both a first line of defence and a bridge to more definitive therapy.

The review also supports the use of TXA in women with BDUC, who comprise a significant proportion of bleeding disorder referrals. As these patients often undergo delivery without a precise diagnosis or individualised management plan, TXA represents a low-cost, accessible, and generally well-tolerated intervention that can be implemented across diverse healthcare settings. Moreover, the safety profile of TXA in this population is reassuring. Across four of the included studies, no thromboembolic events or maternal deaths were reported among 136 women exposed to TXA, echoing findings from larger population-level trials and confirming its favourable risk-benefit ratio in obstetric practice [28,29]. Nonetheless, the strength of these findings must be tempered by an acknowledgment of methodological limitations. All included studies were observational, with most rated as having moderate to serious risk of bias. Several comparator arms were extremely small or poorly matched, raising concerns about confounding and precision. For example, in two of the studies in the meta-analysis, the control arms included only one or two patients, limiting the reliability of direct effect estimates and inflating the risk of Type I error.

The potential for confounding by indication also warrants consideration. Also, phenotype heterogeneity (VWD, FXI, platelet function defects, BDUC) limits generalizability. Co-interventions (DDAVP, factor concentrates, platelet transfusion) and childbirth mode varied across disorders and may have modified apparent effect of TXA, but reporting seldom allowed adjustment. The absence of disorder-stratified or timing-stratified analyses means we cannot specify which phenotypes derive the greatest benefit. In some instances, TXA may have been preferentially administered to women perceived to be at higher risk of bleeding. In contrast, others may have received expectant management based on milder clinical profiles. Without adequate adjustment for baseline characteristics or stratification by severity, the observed associations may be partly influenced by unmeasured variables. Severity differences may also influence therapeutic response. Data on bleeding-disorder severity (e.g., VWF activity, FXI level or platelet function assays) were inconsistently reported across studies and, when reported, often spanned mild to severe defects. Because the antifibrinolytic effect of TXA may differ between severe and mild disorders, this lack of granular severity information represents a key limitation and diminishes our ability to tailor recommendations.

Moreover, the included studies grouped diverse bleeding disorders, which range from VWD and FXI deficiency to BDUC and platelet function abnormalities, without sufficient granularity for disease-specific sub-analyses. This heterogeneity obscures the ability to make tailored recommendations for different patient groups. Compounding this challenge, none of the studies disaggregated outcomes by the timing, dose, or route of TXA administration. It remains unclear whether prophylactic use before delivery, therapeutic use after bleeding onset, or a hybrid approach is most effective for each disorder [29]. Similarly, the effect of concurrent therapies such as desmopressin or clotting factor concentrates was not consistently reported, which limits interpretability.

Despite these gaps, this review builds a compelling rationale for integrating TXA into routine peripartum care protocols for women with diagnosed or suspected bleeding disorders. Diagnostic algorithms developed from the VIBB emphasise the unreliability of single-timepoint factor measurements and underscore the need for standardised testing and follow-up. In practice, however, the opportunity for such evaluation is often missed, particularly in low-resource settings or emergency scenarios. TXA, therefore, offers a rare intersection of efficacy, safety, and feasibility. However, TXA is only one element of postpartum haemorrhage management. Hospital systems and perinatal quality collaboratives have shown that implementation of standardised, stage-based PPH recognition and response protocols improves maternal outcomes and can reduce disparities [30]. Such algorithms emphasise rapid diagnosis, escalation and use of uterotonics and other second-line measures; their success underscores that systematic processes, rather than any single drug, drive haemorrhage control. Given the phenotypic overlap among VWD subtypes, FXI deficiency and BDUC and the physiologic masking of haemostatic defects during pregnancy, conducting finely stratified trials may remain challenging. Nevertheless, embedding TXA within algorithm-driven PPH bundles and multidisciplinary care pathways may maximise its benefit while acknowledging that further differentiation of pathogenic conditions may not be feasible in the real world.

Beyond inherited bleeding disorders, the utility of TXA may extend to hyperfibrinolytic states such as α2-antiplasmin or PAI-1 deficiency, both of which are known to predispose women to mucocutaneous and postpartum bleeding [31,32]. Although these disorders are rare and diagnostically elusive, antifibrinolytic therapy remains one of the few interventions with proven benefit. Given the lack of standardised fibrinolysis assays, empirical TXA use may be necessary and justified in suspected cases. Clinical experience further supports this notion, with studies reporting TXA responsiveness even in patients lacking clearly defined pathophysiology.

Future research should focus on addressing the persistent evidence gaps identified in this review. Chief among these is the need for prospective, controlled trials evaluating the efficacy and safety of TXA in women with clearly stratified bleeding disorders. Such trials should include robust diagnostic algorithms, stratify by disease subtype (e.g., VWD type 1 vs. 2N), and consider patient-level biomarkers such as thrombin generation capacity or platelet function. In parallel, pharmacokinetic studies are needed to determine whether standard TXA dosing adequately accounts for the hemodynamic changes in pregnancy, including plasma volume expansion.

Incorporating biomarker-informed decision-making into clinical practice could refine the use of TXA beyond empirical strategies and enhance its targeted and efficient application. Development of validated assays for thrombin generation, clot lysis time, or tissue plasminogen activity would enhance clinicians’ ability to identify patients most likely to benefit. In addition, emerging evidence from extensive cohort studies suggests that integrating haematology, obstetrics, anaesthesiology, and pharmacy into shared care pathways can optimise the utility of TXA and overall bleeding management [23,28]. The successes reported in multidisciplinary models at high-volume centres should be translated into protocolized approaches adaptable to various healthcare settings.

## 5. Conclusions

TXA appears to reduce the risk of primary PPH on average among women with inherited or unexplained bleeding disorders and was not associated with safety signals in the available cohorts. However, given the heterogeneity of the phenotype, small sample sizes, and co-interventions, these findings support TXA as an adjunct within disorder-specific peripartum plans rather than a stand-alone solution. To move TXA from provisional intervention to standard of care, we require high-quality trials, standardised diagnostic pathways, and interdisciplinary implementation strategies. In a clinical context where delayed or inadequate treatment can cost a life, the impetus to act is ethical and evidence-based. These findings should not mark the end of inquiry, but rather the beginning of a new era of research and policy that centres the population of pregnant women with bleeding disorders in maternal health planning. Nonetheless, our synthesis draws on only six observational studies encompassing heterogeneous bleeding-disorder types and multimodal haemostatic strategies. As such, the certainty of the effect estimate is limited, and caution is warranted when generalising these findings. TXA should be viewed as one component of comprehensive postpartum haemorrhage care, integrated with factor replacement, uterotonics and standardised PPH protocols rather than a definitive therapy.

## Figures and Tables

**Table 1:** Eligibility criteria (PECOS framework).

Domain	Inclusion	Exclusion
Population	Pregnant or postpartum women (≤6 weeks) with any bleeding disorder: VWD, platelet function defects, haemophilia carriership, FXI, PAI-1 deficiency, or unexplained abnormal bleeding	Women without any diagnosed or suspected bleeding disorder
Exposure	TXA is used for the prevention or treatment of PPH, at any dose or route	Other antifibrinolytics without TXA (e.g., ε-aminocaproic acid), or bundled regimens where the role of TXA could not be isolated
Comparator	Placebo, no TXA, or usual care without TXA in the same clinical context	Comparators with concurrent TXA use or co-interventions not separable
Outcomes	Any maternal haemorrhagic outcome: primary/severe/secondary PPH, transfusion, surgical/invasive control, thromboembolic events, maternal death	Studies that reported no maternal bleeding outcomes
Study Design	RCTs, quasi-experimental designs, cohort studies, case–control studies, or case series (≥5 women); published articles and conference abstracts	Reviews, single case reports, editorials, in vitro/animal studies

VWD, von Willebrand disease; FXI, factor XI deficiency; TXA, tranexamic acid; PPH, postpartum haemorrhage; RCT, randomized controlled trials.

**Table 3:** ROBINS-I risk of bias assessment for included studies.

Study (Author, Year)	Confounding	Selection	Classification	Missing Data	Outcome Measurement	Reporting	Overall Judgement
Castle et al., 2022 [23]	Moderate	Low	Low	Low	Moderate	Low	Moderate
Naveed et al., 2020 [20]	Serious	Moderate	Low	Low	Serious	Low	Serious
Berkowitz et al., 2024 [24]	Serious	Moderate	Low	Low	Serious	Low	Serious
Hawke et al., 2016 [11]	Serious	Moderate	Low	Low	Moderate	Low	Serious
Verghese et al., 2017 [22]	Critical	Serious	Unclear	High	Serious	High	Critical
Gerber et al., 2019 [21]	Serious	Serious	Low	Low	Serious	Low	Serious

## Data Availability

The original contributions presented in this study are included in the article and Supplementary Material. Further inquiries can be directed to the corresponding author.

## Supplementary Materials

The following supporting information can be downloaded at: https://www.mdpi.com/article/10.3390/diseases14010034/s1, Table S1: Search Query; Table S2: Excluded studies and reasons for exclusion [4,6,7,8,14,15,16,17,18].
