# Supplementary material for: A Systematic Review and Meta-Analysis on the Effectiveness and Safety of Tranexamic Acid for Postpartum Haemorrhage in Patients with Haemorrhagic Disorders
Source: Diseases. 2026 Jan 19;14(1):34. doi: 10.3390/diseases14010034 (PMC12840432; doi:10.3390/diseases14010034)
Supplement: Supplementary file 1 [file diseases-14-00034-s001.zip › diseases-4076034-supplementary.pdf]

Supplementary material

Table S1: Search Query

Database	Number	Query	Result
**Scopus**	1	("tranexamic acid" OR TXA OR antifibrinolytic*)	27,551
	2	("postpartum hemorrhage" OR "post-partum hemorrhage" OR "postpartum haemorrhage" OR "obstetric hemorrhage" OR PPH)	32,993
	3	"bleeding disorder*" OR "coagulation disorder*" OR "von Willebrand" OR VWD OR hemophilia* OR "platelet function disorder*" OR Glanzmann OR "Bernard-Soulier" OR "factor XI deficiency" OR "factor deficiency" OR thrombasthenia OR "PAI-1" OR "fibrinolytic defect*"	128,446
	1 AND 2 AND 3 AND 4	(TITLE-ABS-KEY ("tranexamic acid" OR TXA OR antifibrinolytic*) AND TITLE-ABS-KEY ("postpartum hemorrhage" OR "post-partum hemorrhage" OR "postpartum haemorrhage" OR "obstetric hemorrhage" OR PPH) AND TITLE-ABS-KEY ("bleeding disorder*" OR "coagulation disorder*" OR "von Willebrand" OR VWD OR hemophilia* OR haemophilia OR "platelet function disorder*" OR Glanzmann OR "Bernard-Soulier" OR "factor XI deficiency" OR "factor deficiency" OR thrombasthenia OR "PAI-1" OR "fibrinolytic defect*"))	310
**PubMed**	1 – TXA terms	(tranexamic acid[Title/Abstract] OR TXA[Title/Abstract] OR antifibrinolytic*[Title/Abstract])	11,444
	2 – PPH terms	(postpartum hemorrhag*[Title/Abstract] OR "Postpartum Hemorrhage"[MeSH Terms] OR "post-partum hemorrhage"[Title/Abstract] OR "postpartum haemorrhage"[Title/Abstract] OR PPH[Title/Abstract] OR "obstetric hemorrhage"[Title/Abstract])	20,908
	3 – Bleeding-disorder terms	(bleeding disorder*[Title/Abstract] OR coagulation disorder*[Title/Abstract] OR "von Willebrand"[Title/Abstract] OR VWD[Title/Abstract] OR hemophilia*[Title/Abstract] OR haemophilia*[Title/Abstract] OR "platelet function disorder"[Title/Abstract] OR Glanzmann[Title/Abstract] OR "Bernard-Soulier"[Title/Abstract] OR "factor XI deficiency"[Title/Abstract] OR thrombasthenia[Title/Abstract] OR "plasminogen activator inhibitor-1"[Title/Abstract] OR PAI-1[Title/Abstract] OR "fibrinolytic defect"[Title/Abstract])	66,579

	1 AND 2 AND 3	Combine the three blocks with AND (no extra filters)	65
**Web of Science (Core Collection)**	1	TS=(tranexamic acid OR TXA OR antifibrinolytic*)	28,458
	2	TS=(postpartum NEAR/1 hemorrhag* OR obstetric hemorrhag* OR PPH)	43,914
	3	TS=(bleeding disorder* OR coagulation disorder* OR von Willebrand OR VWD OR hemophilia* OR haemophilia OR platelet function disorder* OR Glanzmann OR Bernard-Soulier OR "factor XI deficiency" OR factor deficiency OR thrombasthenia OR PAI-1 OR fibrinolytic defect*)	421,348
	1 AND 2 AND 3	Combined	210
**Dimension**	1	("tranexamic acid" OR TXA OR antifibrinolytic OR antifibrinolytics)	22,687
	2	("postpartum hemorrhage" OR "post-partum hemorrhage" OR "postpartum haemorrhage" OR "obstetric hemorrhage" OR PPH)	40,026
	3	("bleeding disorder" OR "coagulation disorder" OR "von Willebrand" OR VWD OR hemophilia OR haemophilia OR "platelet function disorder" OR Glanzmann OR "Bernard-Soulier" OR "factor XI deficiency" OR "factor deficiency" OR thrombasthenia OR "PAI-1" OR "fibrinolytic defect")	122,196
	1 AND 2 AND 3	("tranexamic acid" OR TXA OR antifibrinolytic OR antifibrinolytics)AND("postpartum hemorrhage" OR "post-partum hemorrhage" OR "postpartum haemorrhage"OR "obstetric hemorrhage" OR PPH) AND ("bleeding disorder" OR "coagulation disorder" OR "von Willebrand" OR VWD OR hemophilia OR haemophilia OR "platelet function disorder" OR Glanzmann OR "Bernard-Soulier" OR "factor XI deficiency" OR "factor deficiency" OR thrombasthenia OR "PAI-1" OR "fibrinolytic defect")	111

Table S2: Excluded studies and reasons for exclusion

S/N	Author (year)	Study design & setting	Bleeding-disorder cohort	TXA use & context	Comparator / reference	Main data reported	Protocol-based reason for exclusion
1	(Veen et al., 2019) [4]	Retrospective cohort, NL tertiary centre	Women with severe PPH ≥ 2000 mL; 16/85 had a newly diagnosed disorder	TXA given to ~60 % as part of routine PPH package	None (observational description only)	Diagnostic yield of haemostatic work-up; no TXA effect estimate	TXA not isolated; no TXA-free comparator arm
2	(Wilson et al., 2021) [6]	Retrospective case-series, AUS tertiary centre	VWD (23 deliveries)	Only 2 cases received TXA	All other VWD deliveries	PPH, transfusion	< 5 TXA-exposed women ⇒ fails minimum size requirement
3	(Govorov et al., 2016) [7]	Observational report from clinical series	Multiple VWD patients (number not specified)	Always co-administered with DDAVP/VWF concentrate	Not reported	TXA used concurrently; outcomes on bleeding control	TXA effect not isolatable; always combined with other haemostatic agents
4	(Sarıdoğan et al., 2023) [8]	Case report + literature review	Bernard-Soulier syndrome	Platelets + TXA in single case; narrative review	Historical reports	Qualitative trends	Single case ⇒ < 5 women; heterogeneous anecdotal data
5	(Stoof et al., 2015) [14]	National retrospective survey, NL centres	VWD and haemophilia carriers (46 pregnancies)	Post-partum TXA common but not quantified per patient	None (descriptive audit)	Primary and secondary PPH rates	TXA dose/frequency not reported per case; cannot separate TXA impact
6	(Wolf et al., 2020) [15]	Retrospective matched cohort vs healthy controls	Mixed inherited bleeding disorders (46 pregnancies)	TXA sporadic (mostly not given)	Obstetric controls without bleeding disorder	Odds of PPH vs controls	No within-cohort TXA vs no-TXA comparison; comparator lacks bleeding disorder

7	(Kalot et al., 2021) [16]	Systematic review used for ASH guideline	Pooled VWD studies	Review-level comparison	—	Summary RRs for TXA vs no TXA	Secondary research; protocol includes only primary data
8	(Machin & Ragni, 2020) [17]	Retrospective cohort (rVWF vs. pdVWF)	VWD (12 deliveries)	TXA not part of prophylaxis (given only if bleeding)	rVWF vs. pdVWF	PPH incidence, EBL	No TXA exposure group defined; wrong intervention
9	(Prabhakaran et al., 2020) [18]	Single case report (PAI-1 deficiency)	1 pregnancy	Continuous TXA ante- and post-partum	—	Haemostasis monitored with TEG	Single case ⇒ < 5 women
